# Physiologic Changes during Sponge Bathing in Premature Infants

**DOI:** 10.3390/ijerph18052467

**Published:** 2021-03-03

**Authors:** Jongcheul Lee, Yaelim Lee

**Affiliations:** 1 College of Nursing, The Dankook University of Korea, Chungnam 3116, Korea; shiny1219@dankook.ac.kr; 2 College of Nursing, The Catholic University of Korea, Seoul 06591, Korea

**Keywords:** premature infant, bathing, physiologic response

## Abstract

In this study, we observed physiological reactions of premature infants during sponge bathing in the neonatal intensive care unit (NICU). The infants’ body temperature, heart rate, and oxygen saturation were monitored to examine hypothermia risks during bathing. The participants of the study were 32 premature infants who were hospitalized right after their birth in the V hospital in Korea between December 2012 and August 2013. The informed consents of the study were received from the infants’ parents. The infants were randomly assigned into two-day and four-day bath cycle groups and their physiological reactions were monitored before bathing as well as 5 and 10 min after bathing. The collected data were analyzed using the SPSS statistical package through *t*-test. A significant drop in body temperature was noted in both groups; that is, 4-day bathing cycle and 2-day bathing cycle (*p* < 0.001). However, there were no significant changes in heart rate or transcutaneous oxygen levels. There was no significant change between groups at each measurement point. In order to minimize the physiological instability that may be caused during bathing, the care providers should try to complete bathing within the shortest possible time and to make bathing a pleasant and useful stimulus for infants.

## 1. Introduction

The number of premature infants born before 37 weeks of gestational age is estimated to be almost 15 million [1,2], and their health problems have been reported worldwide [1,3,4]. The advances in medical intervention and treatment have improved the survival rate of premature infants in recent years [5]. Moreover, in Korea, the survival rate of very low birth weight (VLBW) infants (i.e., weight less than 1500 g) significantly increased to 85.7% in the 2010s compared with 83.0% observed in the 2000s. During a similar period, a significant increase in survival rate was observed among extremely low birth weight (ELBW) infants (i.e., weight less than 1000 g), which increased from 66.1% to 70.7% [6]. However, there still are risks of developing short-term and long-term complications [7].

Complications of premature infants were the leading cause of death in children younger than 5 years old globally in 2016, accounting for approximately 16% of all deaths, and 35% of deaths among newborn infants [8]. In Korea, approximately 2500 VLBW infants requiring neonatal intensive care are born every year [9]. Prematurity is one of the most important reasons for hospitalization in the neonatal intensive care unit (NICU). NICU is where premature infants stay from several weeks to months, also known as the period when their central nervous system consistently develops [10]. Hospitalized premature infants are often exposed to stimulants including temperature, touch, light, sound, and smell that are very different from those they have experienced in utero. While they receive life-saving medical treatment and nursing care, they are exposed to numerous stressors such as frequent nursing activities [11] and environmental stimulants like repetitive and excessive noise, light, medical intervention, needle stick, bathing, suctioning, feeding, position changing, painful stimuli, and diagnostic tests [12]. These can affect the infants’ cognitive development by impacting the development of the central nervous system. For example, the noise in NICU was louder than in home and work environments. This intense noise affected infants’ heart rates (causing both bradycardia or tachycardia) and increased their heart rate [13]. Moreover, several studies have explored the environmental factors of NICU that can negatively affect infants’ sleep [14]. Considering the prematurity of the infants, these stimulants can cause potential complications in the long term [11,15,16]; therefore, it often becomes a dilemma for NICU caregivers [10].

Among various interventions, bathing is one of the routinely conducted nursing tasks performed in NICU essential for the promotion of hygiene and neurophysiological development [17]. Although bathing provides many benefits such as the elimination of pollutants from the skin, and thus the prevention of infections, for premature infants, despite triggering and adverse physiological response [18], bathing induces stress, and thus has negative physiological and behavioral influences like increasing heart rate and cardiac oxygen demand [11,17,19]. In particular, in contrast to full-term infants, premature and VLBW infants have an incompletely developed thermoregulatory system, making them highly vulnerable to changes in environmental temperature [20]. In premature infants who have poor temperature alteration [21,22,23], bathing can cause hypothermia [24,25] and the instability of temperature can occasionally cause complications such as hypoglycemia, apnea, hypoxemia, neurologic disorders, acidosis, pulmonary insufficiency, and hemorrhage, thus hypothermia is a major contributor to newborn mortality worldwide and remains a common problem for VLBW infants, even in technologically advanced hospital setting [26].

NICU nurses can conduct sensitive observations and interventions to minimize the premature infants’ exposure to excessive stimulants that can cause stress and potential risks. In this study, we explored the premature infants’ physiological responses to bathing, focusing on their changes in temperature, heart rate, and transcutaneous oxygen saturation. These responses were compared between two groups: 2-day bathing and 4-day bathing groups. We hypothesized that these changes vary widely in 2-day compared with 4-day bathing groups. Each participants’ physiological responses were measured before bathing as well as 5 min and 10 min after bathing. Each group received eight baths, leading to 24 measurements (i.e., 3 measurements each for 8 baths). The mean values of the eight measurements at each assessment point were used in this study.

## 2. Materials and Methods

### 2.1. Participants and Settings

This study was conducted after receiving approval from the institutional review board of the V hospital (VC12OASI0168). According to a preliminary study on the current 2-day bathing group, a standard deviation of 0.26 points before and after the intervention was assumed as the merged standard deviation of the differences between the two groups. Assuming a non-inferiority limit of 0.3 points, and setting the unilateral significance level to a power of 80%, the number of subjects in each group was 12. Considering the drop-out rates of 20% from the preliminary study, we targeted 16 subjects in each group. The participants were 32 premature infants born in V hospital of the C Univ. of Korea and hospitalized in NICU between December 2012 and August 2013.

The inclusion criteria of the subjects were premature infants younger than 37 weeks of gestational age, who were considered by the medical team to have a stable enough general condition and vital signs to take a bath, and written consent was received from their parents, who understood the purpose of this study. The infants who were excluded from the study were those who were unable to receive bathing, for example, those receiving mechanical ventilator care, having intraventricular hemorrhage, having a skin injury, or medically deconditioned.

### 2.2. Intervention

The room temperature before bathing was kept at 26 °C, and the bathwater temperature was kept at 40 °C right before bathing using Thermofinder FS300 (HubDIC Co., Anyang, Korea). The bathing time was 16:00~17:00, ordinarily, and was conducted by a registered nurse who had worked at NICU for over 2 years. The bathing time was set at 7 min and measured using Stopwatch C-516 (Chung’s Electronic Co., Ltd., Hong Kong, China). Other necessary supplies were sterilized puspan; 25 pieces of 10 × 10 cm sized sterile gauze (HP-K-95, Dasom, Nantong, China); 0.5 L of sterile distilled water (Daehan Pharm Co., Ltd., Ansan, Korea); and two pairs of disposable sterile gloves, diaper, towel, and cap.

The standardized bathing method was taught to all nurses in NICU before the study was initiated. The conductor performed hand hygiene before preparing supplies, and removed the hat and diaper. After washing hands again, the conductor put on disposable sterile gloves. Using the sterile gauze wet with sterile distilled water after checking the water temperature, the conductor washed different parts of the body in a cephalocaudal direction in the incubator (i.e., order of face, eyes, neck, chest, back, axilla, limbs, genitals, and bottom). Then, dry gauze was used to dry the scalp and axilla. Infants’ other body parts were dried using a towel. Their mattress, hat, diaper, and clothes were changed and monitor electrodes were attached to their bodies. Lastly, the infants’ sinuses were wiped using a saline-moistened cotton swab.

### 2.3. Instruments

The clinical data measured in this study were temperature, heart rate, and transcutaneous oxygen saturation. The temperature was measured using an axillary thermometer (Thermoval Rapid Electronic, Hartmann Inc., Heidenhim, Germany). Participants’ heart rate and transcutaneous oxygen saturation were measured twice for 30 s to calculate the average, using an echocardiogram monitor (Marquette Dash 3000, General Electronic Inc., Milwaukee, WI, USA), electrodes (Neotrode 1731-003, Con MED, Utica, NY, USA), and sensors (MAXNI, Covidien, Dublin, Ireland).

### 2.4. Data Analysis

The collected data were analyzed using SPSS (18.0, IBM Co., Armonk, NY, USA) statistics package. The general characteristics of the participants were analyzed using descriptive analysis (i.e., frequency, percentage, mean, and standard deviation). The normality of the data was tested using the Shapiro–Wilk test (*p* > 0.05). The physiological measurements before as well as 5 min and 10 min after bathing were compared using paired *t*-test. All statistical analyses were two-tailed and used a significance level of 0.05, and a *p*-value of less than 0.05 was considered to be statistically significant.

## 3. Results

### 3.1. General Characteristics of Participants

The characteristics of the participants are shown in Table 1. Half of the participants were male infants and the other half were female infants. Their mean gestational age was 32.0 weeks and mean weight at study was 1556.3 g. They were infants whose mean length of stay at hospital was 16.2 days. Most of the participants were delivered by cesarean section (71.9%) and their mean 1 min and 5 min Apgar scores were 5.8 and 7.5, respectively. Moreover, 81.3% of the participants were diagnosed with prematurity only, 15.6% with prematurity and respiratory distress syndrome, and 3.1% with prematurity and persistent pulmonary hypertension of newborn.

### 3.2. Temperature, Heart Rate, and Transcutaneous Oxygen Saturation

Body temperature of infants in both the 4-day bath cycle and 2-day bath cycle showed a significant decrease after bathing for 5 min and 10 min (*p* < 0.001). However, there were no significant differences between groups at each measurement point (Table 2).

The infants’ heart rate tended to decrease as they continued bathing. However, the heart rate did not show a significant difference after bathing for 5 min and 10 min. Moreover, there was no significant difference between groups at each measurement point.

Transcutaneous oxygen levels did not show significant differences after bathing for 5 min and 10 min. There was no difference between groups at each measurement point.

## 4. Discussion

In this study, we explored the physiological changes of infants who received bathing on a 4-day cycle and 2-day cycle. A significant drop in body temperature was noted in both groups. However, there were no significant changes in heart rate or transcutaneous oxygen levels. Moreover, there were no significant changes between groups.

Based on these findings, we pose several suggestions for clinical practice and future research. First, we suggest further studies comparing physiological changes by bathing methods. The findings on the infants’ temperature were consistent with the previous study showing the decrease in body temperature with bathing [23]. However, several studies compared different bathing methods to find out methods to minimize the drop in infants’ body temperature during bathing. A previous study comparing body temperature during swaddled bathing (the majority of the infant’s body would be immersed from the shoulders down) and conventional bathing (under the tub) showed that the body temperature decreased after bathing during swaddled bathing [21]. In Loring et al.’s study, body temperature changes during sponge bathing and tub bathing were compared [23]. Less changes in body temperature were noted in tub bathing than in sponge bathing.

Second, we suggest proper up-regulation of the incubator temperature and humidity during bathing to prevent body temperature loss. Although bathing was performed inside the incubator in this study, still, the infants’ body temperature significantly dropped. Neonatal hypothermia, defined as a temperature <36.5 °C, is a major contributor to neonatal mortality and morbidity [27]. The World Health Organization defined an infant’s temperature of 36–36.4 °C as cold stress and 32–35.9 °C as moderate hypothermia, but moderate hypothermia less than 36 °C as a critical cutoff temperature of newborn infants because the complication rate increases when core temperature is below this target [28]. Hypothermia is a major factor in neonatal mortality worldwide [29,30]. Cold stress and hypoglycemia are the two important problems in late premature infants that require immediate treatment [31]. If body temperature control is poor, it can cause hypoglycemia, hypoxia, metabolic acidosis, and so on by metabolism of brown fat [31]. As a result, weight gain, growth, and development are affected. The exact impact of a decrease in body temperature that recovers after 10 min on the infants’ health is unclear. However, considering the immature epidermal barrier in premature infants and the fact that infants’ core body temperature can easily drop by 2–3 °C within 30 min of birth, it is better to prevent this drop in body temperature [24,31].

Third, careful interpretation is necessary of the findings of heart rate and percutaneous oxygen saturation. According to the previous studies, which observed heart rate, respiration rate, oxygen saturation, and body temperature as physiological parameters during tub bathing and sponge bathing, a greater number of infants’ heart rates (those who received tub bathing) were maintained within the normal range [32,33]. Although it is very difficult to judge whether the results differ depending on the researcher or bathing method, it may be thought that handling during bathing is not an excessive stimulus to cause a negative effect on premature infants. However, premature infants, especially VLBW infants, who have been cared at NICU are vulnerable to various stimuli. Stressful stimulation can be detrimental to health, but an appropriate amount of positive sensory stimulation can be beneficial for emotion and developmental progression throughout the life cycle [34].

Forth, health care providers should consider environmental stress when caring for premature infants [16,34]. When providing bathing to infants, the health care providers’ touch can be tactile of a positive effect rather than a negative effect on the premature infants. More studies and evidence are needed to find out optimal handling methods and period.

The study poses several limitations. Hypothermia of infants can be caused for various reasons including mother’s health before delivery and quality of prenatal care [35,36]. Insufficient prenatal care and untreated mother’s disease (e.g., syphilis) have been shown to cause infant’s hypothermia [35]. These factors are not well considered in this study. Moreover, the generalizability of the findings is limited. Although the National Health Insurance coverage is wide in South Korea, there still exist medically underserved areas or districts that do not have obstetrics and gynecology clinics [37]. These factors can affect the infants’ prenatal care and NICU care, which can both contribute to their hypothermia.

## 5. Conclusions

Clinical interventions that can minimize changes in the physiological responses of premature infants need to be continuously sought. In addition to bathing, by looking back on various nursing situations performed routinely or intermittently in NICUs, various clinical interventions need to be reassessed. In the NICU setting, continuous physiological data can be collected [38]. The health care providers need to consider a soothing experience and muscle relaxation, which can induce more beneficial stimulation to the premature infants. Moreover, proper bathing education and training is necessary for both healthcare providers and parents.

## Figures and Tables

**Table 1:** General characteristics of participants (N = 32).

Characteristics		*N* (%) or Mean ± SD
Gender		
	Male	16 (50.0)
	Female	16 (50.0)
Gestational age (weeks)		32.0 ± 2.8
Post conceptional age (weeks)		33.1 ± 2.1
Birth weight (g)		1553.4 ± 445.1
Weight at study (g)		1556.3 ± 421.2
Age at study (days)		8.5 ± 8.4
Length of stay (days)		16.2 ± 11.2
Delivery type		
	Normal spontaneous delivery	9 (28.1)
	Cesarean section delivery	23 (71.9)
Apgar score (1 min)		5.8 ± 1.4
Apgar score (5 min)		7.5 ± 1.3
Diagnosis		
	Prematurity only	26 (81.3)
	Prematurity + Respiratory distress syndrome	5 (15.6)
	Prematurity + Persistent pulmonary hypertension of newborn	1 (3.1)

SD = standard deviation.

**Table 2:** Comparisons of physiological responses by groups and variables (N = 32).

Variables	*N* (%) or Mean ± SD			*p*
	4-Day Bath Cycle (*n* = 16)	2-Day Bath Cycle (*n* = 16)	*t*	
Body temperature				
Before	36.6 ± 0.2	36.6 ± 0.1	0.000	0.738
After 5 min	36.2 ± 0.2	36.2 ± 0.2	0.000	0.799
Change from “Before”	−0.4 ± 0.2	−0.4 ± 0.2	0.000	0.595
*p*	<0.001	<0.001		
After 10 min	36.3 ± 0.2	36.4 ± 0.1	−1.789	0.127
Change from “Before”	−0.3 ± 0.2	−0.2 ± 0.1	−1.789	0.205
*p*	<0.001	<0.001		
Heart rate (beats/min)				
Before	148.6 ± 8.6	143.4 ± 9.4	1.633	0.113
After 5 min	146.9 ± 9.5	143.5 ± 9.9	0.991	0.332
Change from “Before”	−1.7 ± 7.0	0.1 ± 6.4	−0.759	0.447
*p*	0.347	0.938		
After 10 min	145.5 ± 9.7	140.7 ± 8.3	1.504	0.142
Change from “Before”	−3.1 ± 8.6	−2.7 ± 5.5	−0.157	0.884
*p*	0.175	0.068		
SpO_2_				
Before	98.7 ± 1.8	98.4 ± 1.7	0.485	0.623
After 5 min	98.8 ± 1.1	98.6 ± 1.3	0.470	0.653
Change from “Before”	0.1 ± 0.6	0.2 ± 2.1	−0.183	0.848
*p*	0.875	0.722		
After 10 min	98.7 ± 1.4	99.1 ± 1.1	−0.899	0.321
Change from “Before”	0.0 ± 1.2	0.8 ± 1.5	−1.666	0.134
*p*	1.000	0.068		

SpO_2_ = percutaneous oxygen saturation.

## Data Availability

The data presented in this study are available on request from the corresponding author. The data are not publicly available due to the informed consent statement.
